# Supplementary material for: Computerized neuropsychological assessment in post-COVID condition
Source: PLoS One. 2025 May 9;20(5):e0322304. doi: 10.1371/journal.pone.0322304 (PMC12064017; doi:10.1371/journal.pone.0322304)
Supplement: Attachment — Submitted filename: Review_PlosOne_Responses_APA.docx [file pone.0322304.s002.docx]

**RESPONSES TO THE EDITOR AND REVIEWERS’ COMMENTS**

**Dear Editor and Reviewers,**

**Attached you will find the revised version of our manuscript “Computerized neuropsychological assessment in post-COVID condition” (**PONE-D-24-46762)**.**

**We thank the editor and reviewers for their interesting observations and contributions to our article, and the opportunity to submit a revised version of the paper. We address each of their commentaries below:**

**Editor:**

**Thank you for addressing the reviewers' comments in a revised version while focusing on reviewer 3 and reviewer 1 comments. In addition, please provide greater details regarding how PCC (WHO) definition was exactly applied in the PCC sample. That is for each individual how the WHO was applied on each relevant criterion of the definition and using which tools.**

**Response:** The diagnosis of PCC followed the recommendations and definition provided by WHO (Soriano et al., 2022). It is based on the examination and observation of the patients, paying attention to the evolution and the symptoms. Currently, there are no validated diagnostic tests for PCC.

We only included patients who met the criteria according to indications, excluding those with any neurological disorders involving cognitive impairment or other systemic or psychiatric conditions that could potentially impact the findings. All participants in our study also underwent neuroimaging tests (MRI in all cases) to exclude other causes. Additionally, CSF biomarkers of neurodegeneration and FDG-PET were conducted whenever there was even minimal suspicion of a neurodegenerative process.

We have completed the Methods section specifying these aspects.

**Was fatigue measured at any stage? And other key post-viral syndromes? Was this also completed in the health controls? How comorbid causes were excluded (or not) as explanatory (or not) of the current post-viral symptoms as required by the WHO definition? Was a clinical examination conducted and if so by whom and using which instruments?. This additional information is needed to establish the robustness of the PCC definition in the current study.**

**Response:**

Fatigue was assessed at the time of assessment using the Modified Fatigue Impact Scale (MFIS).

Post-viral syndromes were ruled out taking into account the patient's medical history. Patients with a previous post-viral syndrome were specifically excluded.

For patient selection, both physical and neurological examinations were performed. The psychical examination also facilitated the assessment of potential organ damage. Additionally, complementary tests such as blood tests, X-rays, and brain MRI scans were conducted when necessary to exclude other underlying causes. In addition, to exclude other potential causes (such as psychiatric disorders, post-viral syndromes) all patients were interviewed, and the information was cross-checked with the hospital and regional medical chart.

Following the Editor’ suggestions, we have completed the Methods section accordingly.

**Furthermore, please provide additional information regarding acute infection including, how long after acute COVID infection, were the participants evaluated? What types of SARS-CoV-2 variants were most prevalent at the time of this study (by participants of these data is available), how many were vaccinated, how many have received antiretrovirals? Include these as limitations if these data were not available and discuss how this may have impacted the results.**

**Response:**

Patients were evaluated 17.59 ± 8.86 months after infection.

The specific type of SARS-CoV-2 variants was not available in our study, but we had information about the waves based on the date of infection of the patients. Based on data from the Spanish Ministry of Health (2024), the first wave appeared between March 2020 and May 2020 (first strain predominated); the second wave occurred between August and November 2020 with the alpha variant (the B.1.1.7), the third wave occurred between December 2020 and July 2021 with the delta variant (the B.1.617.2), and the last wave, from December 2021 to the present, with the predominance of the omicron variant (B.1.1.529). (These data come from the Spanish Ministry of Health (2024); Spanish Ministry of Health, “SARS-CoV-2 disease in Spain”. Updated July 11, 2024). (Data from the Spanish Ministry of Health, 2024).

Based on the first date of symptom onset of the acute infection was used to determinate the waves of infection: one hundred forty patients (61.7%) belonged to the first wave (March-May 2020), thirty-eight (16.7%) to the second wave (alpha variant, August-November 2020), seventeen (7.5%) to the third (delta, December 2020-July 2021), and thirty-two (14%) to the fourth or subsequent waves. We have completed the manuscript with this information.

Regarding the vaccination status: 221 (96.4%) patients were not vaccinated on the first date of infection. At the time of cognitive assessment, 156 (68.7%) patients were vaccinated.

Treatment received with antiretrovirals during the acute phase was not included in the analysis because the information was not available for all the patients. However, in our sample, only 23.9% of patients were hospitalized. Considering that antiretrovirals were mainly administered to hospitalized patients during the first wave, we believe it is unlikely to affect the results in our sample, given that this represents a small proportion of patients. We have completed the limitations section as suggested.

**Could you also explain why the current study participants were recruited from a Neurology tertiary service? What are the consequences of the current samples’ ascertainment? These data are needed to better establish the representativeness of the sample in comparison to the Spanish general population at the same time of the pandemic. Please include references to relevant Spanish general population data.**

**Response:** Patients were recruited from our hospital and we included patients who consulted due to persistent cognitive issues and other symptoms, such as fatigue, after COVID-19. Madrid, and especially our area, has a healthcare system providing easy access to Neurology. In this regard, although we are a tertiary center providing specialized care, we have direct access from primary care. In fact, we have a direct access from primary care to the neurologists caring for patients with post-COVID, avoiding the bias of selecting the more severe cases. Additionally, the free choice of hospital in Madrid also allows to the patient to select a specific hospital and medical professionals. Patients made their choices based on various factors, such as knowledge of other patients who had been treated, recommendations, specialization, and other considerations. (Matías-Guiu et al., 2015). This allowed us to reach a larger sample size.

The data included in our study are in accordance with previously published studies in the Spanish population. PCC is more prevalent in women, aged 30-55 years and affects patients with both mild and severe forms of the disease (Ledo et al., 2021; López-Sampalo et al., 2022). Moreover, our sample is consistent with other studies carried out in the Spanish population with PCC regarding cognitive symptoms, in demographics and clinical terms (Ariza et al., 2022; García-Sánchez et al., 2022; Serrano del Pueblo et al., 2024).

**Reviewer #1:**

**1-Thank you for allowing me to review this interesting and clinically-needed research Throughout the paper, the term Post COVID condition (PCC) is used. It is unclear if this refers only to persistent cognitive symptoms. Please include how you are defining the term.
Can you please describe why 6 months was chosen for persistent symptoms, as opposed to 3 months which is the definition of PCC.**

**Response:**

Thanks for all the positive comments and suggestions to improve the paper. Post-COVID Condition (PCC) refers to the condition defined by the WHO (WHO, 2021). This condition occurs in individuals with a history of SARS-CoV-2 infection approximately 3 months from the onset, and symptoms lasting for at least 2 months, cannot be attributed to alternative diagnoses (Soriano et al., 2022). The diagnosis is based on the examination and observation of the patients, paying attention to the evolution and the symptoms. Currently, there are no validated diagnostic tests for PCC.

Patients were selected with a 6-month criterion. The 6-month criterion was used to separate possible symptoms that may occur after the acute or subacute phase of the disease (inflammation) and persistent symptoms. This criterion was established to be able to separate those possible cases where there may be a spontaneous recovery and to recognize those patients in whom the symptoms have become chronic (Correa & Vallespín, 2022). In other similar pathologies such as Chronic Fatigue Syndrome (CFS), the 6-month criterion is also used to define this condition as chronic (Fernández et al., 2009). We have completed the manuscript with this information.

**2-Could the HC group include other forms of Long COVID? For instance, for people who report post-covid persistent changes in smell perception, what group would they go into?**

**Response:** The recruitment of control subjects was carried out through the Neurology Department by reaching out to patients’ relatives, caregivers, and friends, as well as in collaboration with neighborhood associations in Madrid and nearby areas. Participants were asked about their COVID-19 infection history, and a questionnaire on potentially associated symptoms was administered to confirm that these individuals did not experience any symptoms following the infection. We have completed the Methods section accordingly.

**3-Methods: Can more detail be provided on the administration of the measures. Were they done in person or remotely? What was the role of the administering neurologists? Did they read instructions and clarify? Can discussion of whether the battery could be used remotely be added? Has the Vienna Test System been validated? Has it been shown to correlate with other neuropsychological measures?**

**Response:** The computerized battery was administered in person at our hospital by trained neuropsychologists. It was conducted in two-day sessions, following the battery guidelines. Each session lasted approximately 80 minutes. Before testing, the neuropsychologist provided a summary of the tests’ aims, duration, and content of testing. Participants were also informed about how data would be used (e.g. anonymization or documentation). Each participant was required to sign the informed consent form and was reminded to use their glasses and hearing aids, if necessary. The instructions and procedures are standardized, and during test administration, the administrators were instructed not to interfere with the instructions, as participants were expected to work independently (ideally without assistance). However, the neuropsychologist could provide assistance to resolve any issues or doubts. Another important aspect involved observing and recording the participant's conduct and behavior during the test.

We have included more details in the Methods section.

Regarding the normative data, the tests battery has several standardization studies in different countries and different samples (adults, children’s and different professional samples) (Aschenbrenner et al., 2012; Sturm, 2018). More details are explained below:

For the COGBAT Index: the norm sample for the standard form was collected (2012-2013) from the research laboratory at SCHUHFRIED GmbH and in cooperation with the teaching and research activities at the University of Vienna. Norming process involved a comprehensive sample of normal people that in terms of gender and age was representative of the general population of Germany, Austria, and Switzerland the data relates to an Austrian sample of 419 individuals from the normal population aged between 16 and 80. The sample consists of 184 (44%) men and 235 (56%) women. Participants were only included if they could state that they had not previously suffered from any serious neurological or psychiatric illness. Another representative norm sample was collected in Italy. The representative Italian norm sample was collected at multiple locations in Italy on different occasions. The samples of all tests correspond to the age and gender distributions gained from the demographic data from Italy from 2012 (Aschenbrenner et al., 2012).

Regarding WAF battery: the data of the representative norm sample of 306 individuals was gathered in 2016-2017 in the Test and Research Center of SCHUHFRIED GmbH, using a quota plan stratified according to gender, age and education. The expected age and gender distribution was obtained from demographic data for Germany, Austria, and Switzerland for 2016 (European Commission, 2016). The norm sample consists of 157 (51%) women and 149 (49%) men aged between 14 and 88 (Sturm, 2018). Neglect add-on subtest of the WAF and FLEI add-on were normed separately. The norm data was collected in 2011 in the research laboratory of SCHUHFRIED GmbH. The sample involves 329 respondents taken from the normal population. The sample consists of 151 (46 %) men and 178 (54 %) women. The respondents range in age from 16 to 83 (Beblo et al., 2012).

Other independent tests also have normalization studies. For example, DT: The data of the representative norm sample of 759 individuals was gathered in 1999 - 2017 in the Test & Research Center of SCHUHFRIED GmbH, using a quota sample plan stratified according to gender, age, and education in multiple collection waves. The data of the latest data collection wave was collected from 2016 to 2017 (Schuhfried, 2020); COG: the data of the representative norm sample of 662 individuals was gathered in 2005 - 2015 in the Test & Research Center of SCHUHFRIED GmbH. The data for the norming were collected in two data collection samples between 2005-2007 and in 2015 (Schuhfried, 2013); RT: the representative norm sample of 673 individuals was gathered from 2014 to 2016 at several locations in Italy using a quota sample plan stratified according to gender and age. The expected age and gender distribution was obtained based on demographic data for Italy for 2011 (Istituto Nazionale di Statistica, 2011). The norm sample consists of 311 (46%) men and 362 (54%) women aged between 16 and 94. There are studies in different countries (Slovakia, Brazil, Poland, Netherlands, France…) and with different samples such as professional drivers, Traffic psychological clients, athletes, machine operators (Schuhfried, 1996).

Correlation with other measures:

VTS has demonstrated validity and reliability comparable to traditional neuropsychological measures. Many tests are designed to assess functions, such as attention, memory, processing speed, executive function, and motor skills, and are often compared with other well-established neuropsychological tests.

Some examples of this: TMT-L was compared with the performance of the paper-and- pencil version from Reitan (1992) to test the construct validity of the TMT-L and significant correlations were found for all pairs of variables at the 0.01 level (Rodewald et al., 2012); FGT has shown correlations with a number of other tests, including tests of attention and various executive functions, have been investigated; the results provide evidence of the test’s construct validity (Vetter, J.; Aschenbrenner, S. & Weisbrod, 2018); DT: had shown correlation analyses with a number of further tests from the Cattell-Horn- Carroll model (CHC model), which are often applied internationally in the framework of traffic psychological investigations, prove construct validity of the test’s characteristic interpretations. Furthermore, there is a large number of studies in the areas of traffic psychology, safety assessment, sports and clinical (NEURO) psychology which prove the criterion validity of DT in different application contexts (Schuhfried, 2020); COG: Based on the CHC model (Schneider & McGrew, 2018) the convergent and discriminant validity for the COG was tested with a number of further tests *(LVT, VISGED, ATAVT, Corsi, DT, RT)* (Schuhfried, 2013); RT: RT has the highest correlation with the COG and DT tests as can be expected from the nomological network or the CHC model in which both tests load on all three tests on the secondary factor Reaction & Decision Speed (Gt). Furthermore, the impact of working memory (Gwm) on working the RT is shown which becomes apparent via the correlation with the CORSI. The discriminant validity was tested based on the correlations of the RT with the ATAVT, LVT, and VISGED tests (Schuhfried, 1996).

**4-Edits for clarification:**

**Page 4 last paragraph: But not necessarily all the tests have the same relationship strength with their factors (metric invariance or weak invariance).**

Following the Reviewer’s comment, we have clarified the concept of metric invariance/ weak invariance by providing an example. Thank you for this suggestion.

*“But not necessarily all the tests have the same relationship strength with their factors (metric invariance or weak invariance). For instance, the strength of the relationship between specific measures of attention and the attention factor may vary between groups”.*

**Reviewer#2:**

**Dear Sir/Mam. Please find bellow the requested review regarding the manuscript. The article contains a lot of useful information on the issue. The topic is very interesting and use of sources is appropriate. Although it has some useful information there are less references and the statements are not established. I suggest the authors to write more information with references.**

**The article contains a lot of useful information on the issue. It is quite clear what is already known about this topic and the research question is clearly outlined. The abstract is good and introduction section involves too much information. The introduction should be briefer and the authors should include the studies of:**

**-Megari, Kalliopi, Evanthia Thomaidou and Electra Chatzidimitriou (2024). Highlighting the Neuropsychological Consequences of COVID-19: Evidence from a Narrative Review. INQUIRY: The Journal of Health Care Organization, Provision, and Financing, Volume 61: 1 –10. DOI: 10.1177/00469580241262442**

**- Akyllina Despoti, Kalliopi Megari, Anna Tsiakiri, Maida Toumaian, Vasiliki Koutzmpi, Athanasia Liozidou, Angeliki Tsapanou. Effectiveness of remote neuropsychological interventions: a Systematic Review. Applied Neuropsychology Adult 27:1-9. doi: 10.1080/23279095.2024.2382814.**

**The research question is justified clearly, given what is already known about the topic. The results are not discussed from multiple angles and conclusions answer the aims of the study partially. The conclusions are partially supported by references or results and the limitations of the study fatal and it is questionable if there are opportunities to inform future research. Positive: There are some strengths of the article that could have an impact in the field, such as the topic and its impact on the existed literature.**

**Response:** Thanks for all the positive comments and suggestions. We have added the references mentioned by the reviewer, which we believe that are useful to improve the Introduction. We have also reduced the length of the introduction as suggested. We have also completed the Discussion to improve it. Additionally, we have introduced some comments for future research.

**Reviewer#3:**

**1-Introduction: Page 3, paragraph 1 and 2 refers to “cognitive symptoms” is unclear whether this is self-report or performance based. It would be helpful to have them labeled more specifically (e.g., self-reported cognitive symptoms and Neuropsychological test performance/impairments on neuropsychological tests) for the introduction and also discussion.**

**Response:** Thanks for this comment. We have distinguished and included “Subjective cognitive complaints” and “Objective cognitive impairment”, following the reviewer’ suggestion. We have also clarified this distinction in the Methods section.

**I think it would be helpful to add that the majority of post COVID studies have been using pencil and paper comprehensive evaluations. Since these are not mentioned until paragraph 4, it seemed like these were used less often than the other approaches mentioned in paragraph 3. “However, cognitive screening tests do not allow an in-depth description of the cognitive processes underlying cognitive impairment, and dementia screening tests might not be suitable for people with PCC, whose deficits require tests with more sensitivity than people with dementia.” This sentence would be more helpful with specific reasons: e.g., subtle deficits that may not be detected with less sensitive measures, wider range of premorbid abilities than populations the tests were normed on, wider range of age ranges. More detail on this would help the battery feel more specific for post COVID.**

**“In addition, conducting a cognitive assessment in uncontrolled settings could bias the results obtained”. More detail is needed here. Is this referring to telephone or internet assessments?**

**Response:** Thanks for this suggestion. We have modified the Introduction section as suggested.

**The introduction felt very long in the second half and information page 4 paragraph 2 and 3 seemed like they could fit better in the methods section.**

**Response:** Thanks for this suggestion. We have amended the Introduction and Methods sections as suggested.

**The introduction could be stronger if more sections focused on the specifics of why this is the appropriate next step for post COVID condition, versus just in general. This would be really helpful in page 3, paragraph 4, and page 4 paragraph 1.
o Subtle attentional/processing speed deficits may be detected easier with computerized testing. Why is this relevant to long COVID? Some of the literature on deficits being subtle in this area may be really helpful and justify this measure.**

**Response:** Thanks for this comment. We have completed the Introduction as suggested and included more details about the relevance of these measures.

**2**-**Methods: Was this sample recruited from patients? Or advertised through the Department of Neurology? As neurological disorders were exclusionary, were these patients who reported neurological symptoms but did not meet criteria? Were the 508 participants meeting criteria and enrolled a small percentage of patients seen in the clinic? I feel like this would be a different sample than a community healthy control and more detail would be helpful (in the appendix if not space in the main paper).**

**Response:**

The sample of patients with PCC represents a representative sample of the patients seen, because we included almost all patients attended in this period after checking the inclusion and exclusion criteria. The sample is relatively large and covers the whole spectrum of the disease (hospitalized and non-hospitalized patients, patients with mild-to-moderated symptoms, etc.). Moreover, the sample meets a wide range of demographic (age or sex) and clinical characteristics. It is also comparable with other samples in terms of prevalence and characteristics (explained in the previous section).

Regarding the control group, subjects were recruited from the Department of Neurology, and in collaboration with associations in Madrid and nearby areas. The control group consisted of healthy people without neurological or psychiatric disorders. This group was formed by a large sample, matched with the PCC group in terms of age and years of education.

The rigorous selection criteria as well as the adjustment for clinical and demographic variables guarantee the representativeness of the sample.

We have completed the manuscript adding some comments about this.

**Did neurological disorder include minor neurocognitive disorder or mild cognitive impairment?**

**Response:** We did not include any minor neurocognitive disorder or mild cognitive impairment, because they are well-known disorders potentially associated with prodromal neurodegenerative diseases. We have completed the Methods section specifying it.

**You use cognitive complaints in this section and cognitive symptoms in the introduction. Using the same language for self-reported symptoms and objective performance based impairments throughout the article would be helpful.**

**Response:** We have changed and used consistent names for **“**Subjective cognitive complaints” and “Objective cognitive impairment”. Thanks for this suggestion.

**Statistical methods felt very clear to me and detailed enough to be replicated.
Response:** Thanks for this positive comment.

**3-Results: On page 8, you talk about hospital and ICU admission. It would be helpful to have more detail in the methods section about how you determined this and where you obtained the information.**

**Response:** Thanks for this comment. Information about UCI admission was obtained through patients interviews and corroborated with the hospital’s medical records. This information has been added into the methods section.

**4-Discussion:**

**-The whole paper and discussion would benefit from making the aims of the paper a bit more clear. Is the primary aim to explore the factor structure in a combined sample of PCC and HC with a secondary aim of seeing the factor structure in the PCC group? Or is the primary aim exploring the factor structure in the combined sample and then using the computer testing to examine PCC specific deficits? I think this would be more clear to readers if you used more explicit language about the primary and secondary aims.**

**Response:** Thank you for the suggestion. We have better specified the primary and secondary objectives to clarify it.

**- On page 10, you mention additional adjustments in the WAF divided in RT reaction time. I did not see this mentioned in the results section.**

**Response:** Thank you for this comment. We meant to say that the partial invariance was met with the exception of those tests (in addition to the exception of RT-motor for metric invariance). We apologize for the misunderstanding. We have modified the text accordingly.

**“***With the exception of WAF-divided and RT-reaction scores, the VTS battery showed partial scalar invariance when the model constraint for equivalent test intercepts across groups for all indicators. WAF-divided and RT-reaction indicators were linked to the Attention Processes factor similarly in both PCC and HC groups. However, intercepts differed, and observed scores would vary at different levels of Attention Processes***”**

**- For previous studies highlighting attentional deficits, it would be helpful to have all domains compared, not just attention. Also, it would be helpful to note if they only measured attention. This is touched on a bit with memory but more detail would be helpful.**

**Response:** Many thanks for this comment. We have modified the text accordingly.

*“Previous studies have highlighted attentional deficits in PCC (4,5) and have suggested attentional deficits as a characteristic feature of PCC (8,9) when other cognitive domains, such as memory, executive function, language, and visuospatial skills are also studied. Attention has been previously reported as the most frequent cognitive domain impaired, followed by episodic memory, executive function, visuospatial function, and language”.*

**-Was COVID infection severity similar in the articles you cite with similar findings?**

**Response:** COVID infection severity was similar in other articles. Most of the patients included in our study had mild stages of the disease. In accordance with studies showing that cognitive deficits are more prevalent in people with mild and moderate stages of the disease (Zhao et al., 2023). However, we also have a group with more severe acute disease, thus covering the whole spectrum of the disease, which is important for some of the analyses.

- **For page 10, you state visual memory may be less sensitive than verbal episodical memory tests. It would be helpful to have supporting literature from other test batteries or other populations to bolster this claim.**

**Response:** Thank you for the comment. We have added several references to support this statement.

-**Were there performance validity tests included? If not, this should be listed as a limitation in the discussion section and something that could potentially be added to future studies.**

**Response:** We did not include performance validity tests. However, it would be interesting to include these tests in future studies. This information has been added as a limitation in the discussion. Thanks for this suggestion and idea for future research.

**References:**

Ariza, M., Cano, N., Segura, B., Adan, A., Bargalló, N., Caldú, X., Campabadal, A., Sala-llonch, R., Barrué, C., Bejar, J., Group, N. C., & Junqué, C. (2022). *Neuropsychological impairment in post-COVID condition individuals with and without cognitive complaints*. *October*, 1-12. https://doi.org/10.3389/fnagi.2022.1029842

Aschenbrenner, S., Kaiser, S., Pfüller, U., Roesch-Ely, D., & Weisbrod, M. (2012). Testset COGBAT. *Schuhfried: Mödling*.

Beblo, T., Kunz, M., Albert, A., & Aschenbrenner, S. (2012). Vienna test system (VTS): Mental ability questionnaire (FLei). *Schuhfried: Vienna, Austria*.

Correa, E. M., & Vallespín, G. T. (2022). COVID persistente. Elementos básicos para el médico de atención primaria. *FMC-Formación Médica Continuada en Atención Primaria*, *29*(9), 481-489.

Data from the Spanish Ministry of Health. (2024). *SARS-CoV-2 disease in Spain*.

Fernández, A. A., Martín, A. P., Martínez, M. I., Bustillo, M. A., Hernández, F. J., Jde, L., Penas, R. D., Rivas, E. G., Delgado, C. P., & Giménez, J. R. (2009). Chronic fatigue syndrome. Summary of the consensus document. *Atencion Primaria*, *41*(10), e1-5.

García-Sánchez, C., Calabria, M., Grunden, N., Pons, C., Arroyo, J. A., Gómez-anson, B., Lleó, A., Alcolea, D., Pomar, V., Belvís, R., Domingo, P., Morollón, N., & Mur, I. (2022). *Neuropsychological deficits in patients with cognitive complaints after COVID-19*. *August 2021*, 1-11. https://doi.org/10.1002/brb3.2508

Ledo, M. del P. R., del Olmo, L. A., Caballero, S. G., & Fernández, S. B. (2021). La persistencia de síntomas de la COVID-19 y su diagnóstico en la primera ola de la pandemia en España. *Medicina general*, *10*(2), 53-59.

López-Sampalo, A., Bernal-López, M. R., & Gómez-Huelgas, R. (2022). Síndrome de COVID-19 persistente. Una revisión narrativa. *Revista clínica española*, *222*(4), 241-250.

Matías-Guiu, J. A., García-Azorín, D., García-Ramos, R., Basoco, E., Elvira, C., & Matías-Guiu, J. (2015). Estudio de la asistencia neurológica ambulatoria en la Comunidad de Madrid: impacto del modelo de libre elección de hospital. *Neurología*, *30*(8), 479-487.

Rodewald, K., Bartolovic, M., Debelak, R., Aschenbrenner, S., Weisbrod, M., & Roesch-Ely, D. (2012). Eine Normierungsstudie eines modifizierten TRAIL making tests Im deutschsprachigen Raum. *Zeitschrift für Neuropsychologie*.

Schneider, W. J., & McGrew, K. S. (2018). The Cattell-Horn-Carroll theory of cognitive abilities. *Contemporary intellectual assessment: Theories, tests, and issues*, 73-163.

Schuhfried, G. (1996). Reaction Test. *Schuhfried: Mödling*.

Schuhfried, G. (2013). Cognitrone. *Schuhfried: Mödling*.

Schuhfried, G. (2020). Determination Test. *Schuhfried: Mödling*.

Serrano del Pueblo, V. M., Serrano-Heras, G., Romero Sánchez, C. M., Landete, P. P., Rojas-Bartolome, L., Feria, I., Morris, R. G. M., Strange, B., Mansilla, F., & Zhang, L. (2024). Brain and cognitive changes in patients with long COVID compared with infection-recovered control subjects. *Brain*, *147*(10), 3611-3623.

Soriano, J. B., Murthy, S., Marshall, J. C., Relan, P., & Diaz, J. V. (2022). A clinical case definition of post-COVID-19 condition by a Delphi consensus. *The Lancet Infectious Diseases*, *22*(4), e102-e107. https://doi.org/10.1016/S1473-3099(21)00703-9

Sturm, W. (2018). Manual Wahrnehmungs-und Aufmerksamkeitsfunktionen-Batterie-WAF (manual perception and attention function battery). *Mödling: Schuhfried*.

Vetter, J.; Aschenbrenner, S. & Weisbrod, M. (2018). Figural Memory Test. *GmbH; Schuhfried & Moedling, Austria*.

Zhao, S., Toniolo, S., Hampshire, A., & Husain, M. (2023). Effects of COVID-19 on cognition and brain health. *Trends in Cognitive Sciences*, *27*(11), 1053-1067. https://doi.org/10.1016/j.tics.2023.08.008
